# Navigating the future—community pharmacists’ attitudes towards AI integration in pharmacy services: a cross-sectional study in Aseer, Saudi Arabia

**DOI:** 10.7717/peerj.21246

**Published:** 2026-05-08

**Authors:** Arwa Khaled, Dalia Almaghaslah, Khalid Orayj, Ayesha Siddiqua, Geetha Kandasamy, Naglaa Samir Bazan

**Affiliations:** 1 Department of Clinical Pharmacy, College of Pharmacy, King Khalid University, Abha, Aseer Region, Saudi Arabia; 2 Critical Care Medicine Department, Cairo University Hospitals, Cairo University, Cairo, Egypt

**Keywords:** Artificial intelligence, Community pharmacist, Pharmacy practice

## Abstract

**Background:**

There have been advances in healthcare technologies in general and in the pharmacy field in particular. Among the most promising is Artificial Intelligence (AI), which has the potential to advance pharmacy practice. The current study was conducted to assess community pharmacists’ attitudes and preferences regarding AI-enabled tools in their day-to-day practice and factors affecting them.

**Methods:**

This study used a cross-sectional self-administered questionnaire. It was conducted in community pharmacies across the Aseer region. A total of 117 community pharmacists were recruited using a non-probability convenience sampling method. The data collection tool was adapted from a previous study. It collected data related to participants’ demographics and their attitudes towards AI-enabled tools in their practice.

**Results:**

A total of 85.5% of the participants had internet access, and 83.8% of them had access to scientific drug information resources. In addition, 81.2% of the participants reported prior awareness of AI. Among those, about half of the participants reported sufficient knowledge of AI (51.6%). A total of 58.9% of them did not think that AI would replace pharmacists in the health care system. However, 71.6% of the pharmacists thought that AI would reduce errors in medical practice. Pharmacists showed preference for using AI in medical data collection, identifying drug-related problems, and medication dispensing.

**Discussion and conclusion:**

Community pharmacists in the Aseer region showed moderate awareness, cautious attitudes, and selective preferences toward AI implementation in pharmacy practice. While pharmacists recognized the potential benefits of AI, limited knowledge and varying levels of digital infrastructure may influence its adoption. These findings highlight the importance of integrating AI education into pharmacy training programs, providing continuing professional development opportunities, and improving digital infrastructure to facilitate the effective implementation of AI technologies in community pharmacy practice.

## Introduction

Community pharmacy has become an increasingly integral component of healthcare delivery in Saudi Arabia (Almaghaslah et al., 2022). Recent restructuring of the national healthcare system, including the digitalization of several services, has expanded the role of community pharmacies in providing pharmaceutical care within the public healthcare sector.

Community pharmacies represent the most accessible healthcare facilities, offering pharmaceutical services on a walk-in basis without the need for appointments, thereby enhancing convenience for the general public (Alsahali et al., 2023). In addition, community pharmacies provide relatively low-cost services, shorter waiting times, and greater opportunities for patient counseling by pharmacists. These attributes have contributed to the growing popularity of community pharmacies in Saudi Arabia (Almanasef, 2023). The increasing demand for community pharmacy services, together with the establishment of public–private partnerships linking government healthcare systems with community pharmacies, has necessitated the adoption of more advanced technologies to support service delivery (Almanasef, 2023).

One such initiative is the Wasfaty system, an electronic prescribing platform that connects government hospitals and primary healthcare centers with private community pharmacies participating in the program (Almaghaslah et al., 2022; Alsahali et al., 2023). The system enables beneficiaries to collect prescribed medications from the nearest participating pharmacy through text message notifications and supports services such as medication re-dispensing and home delivery of medications and medical supplies (Almanasef, 2023). Effective implementation of the Wasfaty system requires community pharmacies and pharmacists to possess specific technical and digital competencies to manage these services efficiently.

The broader digital transformation of healthcare has also involved the introduction of electronic health records, further necessitating that pharmacists acquire advanced digital skills, including competencies related to AI-enabled tools (Raza et al., 2022).

Artificial intelligence (AI) is defined as “the capacity of machines to carry out tasks that ordinarily require human intelligence, including sensing, thinking, learning, and decision-making” (Ranchon et al., 2023).

AI has been widely adopted across healthcare settings, ranging from wearable devices and mobile health applications to sophisticated diagnostic and clinical decision-support systems (Memish et al., 2021). These technologies assist healthcare professionals in clinical decision-making, patient data management, and advanced data processing to support the diagnosis and management of complex medical conditions” (Ranchon et al., 2023).

Other applications of AI in healthcare include electronic patient records, disease diagnosis, surgical assistance, and chronic disease management (Syed & Al-Rawi, 2023b).

With rapid technological advancements in healthcare in general and pharmacy practice in particular, AI has demonstrated a positive impact on various aspects of pharmaceutical care. AI-enabled tools have enhanced drug discovery processes, improved medication management, and strengthened medication safety in a more efficient and systematic manner (Raza et al., 2022). Moreover, AI has enabled pharmacists to shift their focus toward patient-centered clinical activities by reducing the burden of traditional roles such as routine dispensing (Vora et al., 2023).

Although AI has gained increasing acceptance among pharmacists across different practice settings, its integration within the Saudi Arabian healthcare system has provided pharmacists with advanced tools to support accurate, evidence-based clinical decision-making. By leveraging AI algorithms and machine learning techniques, pharmacists can analyze large volumes of patient data—including medical records, laboratory findings, and medication histories—to identify potential drug–drug interactions, evaluate medication safety and effectiveness, and deliver personalized therapeutic recommendations.

Several AI-based models have been developed to predict and detect adverse drug events, support medication-related clinical decision-making, automate dispensing processes in community pharmacies, optimize medication dosing, and enhance medication adherence through smart technologies (Dsouza et al., 2025).

Additionally, AI systems can help detect and prevent medication errors, provide comprehensive medication therapy management services, and support telemedicine initiatives, thereby promoting safer and more efficient pharmaceutical care delivery (Chalasani et al., 2023).

Despite these advancements, community pharmacists’ attitudes and preferences toward the AI-enabled tools have not been sufficiently explored in the Saudi Arabian context. Therefore, the present study was conducted to assess community pharmacists’ preferences and attitudes toward the adoption of AI in community pharmacy practice in Saudi Arabia.

## Methods

### Study design

This study employed a cross-sectional design using a self-administered questionnaire. It was conducted in community pharmacies across the Aseer region.

### Population and setting

The study was carried out among pharmacists practicing in community pharmacies in the Aseer region, which is located in the southern province of Saudi Arabia. According to the most recent data from the Health Statistics Yearbook 2023, the population of the Aseer region is 2,024,285. In Saudi Arabia, there are a total of 23,649 community pharmacists, with 612 practicing in the Aseer region. This corresponds to approximately 3.03 community pharmacists per 10,000 population in the Aseer region (Ministry of Health of Saudi Arabia, 2023).

### Sample size and sampling procedure

The sample size was calculated based on the total number of community pharmacists in the region (*n* = 612) using the Raosoft sample size calculator (https://raosoftcalculator.com/), with a predefined margin of error of 5% and a confidence level of 95%. To minimize erroneous findings and enhance study reliability, the target sample size was set at 237.

A non-probability convenience sampling technique was selected due to its practicality and ease of access to the target population. To partially mitigate selection bias, invitations were distributed to multiple large community pharmacy chains as well as independent pharmacies across different cities and towns in the Aseer region, rather than recruiting from a single organization. In addition, reminders were sent at different time points to capture pharmacists working various shifts.

Despite these efforts, only 117 pharmacists agreed to participate in the study. Participation was voluntary, and informed consent was obtained from all participants prior to enrollment.

### Study period

The study was conducted from November 2023 to March 2024.

### Data collection form

The structured questionnaire was adapted from a previously published instrument developed by Al Meslamani (2023), which examined the use of artificial intelligence (AI) in pharmacy practice. The original survey included items on demographics, AI knowledge, and attitudes. For the current study, core demographic items were retained, including age, gender, nationality, years of experience, working hours, prescription volume, and access to internet and drug information resources. The AI-related sections were adapted to reflect the community pharmacy context in Saudi Arabia.

Specifically, 15 preference items (Section 2) and nine attitude items (Section 3) were retained from the original instrument but reworded to align with community pharmacy services and the local practice environment. Items that focused exclusively on hospital pharmacy or non-applicable technologies (three items) were removed. Additionally, two new preference items addressing the use of AI in insurance and reimbursement processing and social data collection were added to reflect contemporary practice demands in Saudi Arabia.

Section 1 collected demographic and background information, including age, gender, nationality, work experience, working hours, number of prescriptions dispensed per day, availability of internet access, availability of drug information resources, and pharmacists’ knowledge of AI. Section 2 consisted of 15 items measured on a 5-point Likert scale ranging from strongly disagree to strongly agree, assessing pharmacists’ preferences toward incorporating AI into day-to-day practice, including patient counseling, medication dispensing, medication adherence, communication with other healthcare providers, follow-up and patient monitoring, and identification of patient-related problems. Section 3 used the same Likert scale and contained nine items assessing pharmacists’ attitudes toward AI. The questionnaire is provided as an appendix.

The survey was piloted with five community pharmacists who met the inclusion criteria. Feedback from the pilot resulted in minor linguistic and structural refinements, including simplification of technical terminology to enhance clarity and slight reordering of some items to improve logical flow from general to specific AI-enabled tools. No items were added or removed following the pilot; only wording and order were modified. Data from the pilot study were not included in the final analysis.

Face and content validity were assessed by two academic staff members from the Clinical Pharmacy Department at King Khalid University with experience in community pharmacy practice. Each reviewer independently evaluated the questionnaire items for relevance to community pharmacy practice, clarity of wording, and coverage of key AI domains (*e.g.*, clinical decision support, workflow efficiency, and patient care). Suggested modifications, primarily related to item wording and domain coverage, were discussed with the research team and incorporated by consensus to enhance item relevance and clarity.

### Inclusion criteria

Pharmacists were eligible for inclusion if they were licensed in Saudi Arabia, worked in a community pharmacy in the Aseer region, held at least a first degree in pharmacy, and were willing to participate in the study. For the analytical phase, only pharmacists who reported having heard of AI prior to completing the survey were included. This approach was adopted because the preference and attitude scales specifically assessed the adoption of AI-enabled tools, and respondents without prior awareness were unlikely to provide meaningful responses. However, this decision may have introduced selection bias by overrepresenting pharmacists who were already familiar with AI and more engaged with digital technologies.

### Exclusion criteria

Pharmacists and pharmacy technicians working in other pharmacy sectors, including hospitals, industry, academia, and regulatory bodies, as well as those working in community pharmacies located outside the Aseer region, were excluded.

### Statistical analysis

Data were cleaned, coded, entered, and analyzed using the Statistical Package for the Social Sciences (SPSS) version 26.0 for Windows. Demographic, background, and knowledge variables were described using frequencies and percentages. The preference scale consisted of 15 Likert items, and the attitude scale consisted of nine Likert items, each scored from 1 (strongly disagree) to five (strongly agree). Descriptive statistics were presented as means and standard deviations.

For interpretative purposes, mean scores on the 5-point Likert scales were categorized as low (1.00–2.33), moderate (2.34–3.67), or high (3.68–5.00) preference or attitude (Norman, 2010).

Normality of the composite preference and attitude scores was assessed using the Shapiro–Wilk test, skewness statistics, and visual inspection of histograms and Q–Q plots. For the preference scale, the Shapiro–Wilk statistic was *W* = 0.978 (*p* = 0.080), and for the attitude scale, *W* = 0.976 (*p* = 0.050), indicating no significant deviation from normality at the 0.05 level.

Composite scores for the preference (15 items) and attitude (nine items) sections were treated as approximately continuous variables, as they represented the mean of multiple Likert items, demonstrated acceptable normality (Shapiro–Wilk *p* > 0.05 and skewness within ±2), and parametric tests are considered robust and appropriate for multi-item Likert scales in health and social science research.

Independent t-tests and one-way analysis of variance (ANOVA) were applied to examine factors associated with pharmacists’ preferences and attitudes toward AI. Statistical significance was set at *p* < 0.05. Internal consistency and reliability of the scales were assessed using Cronbach’s alpha coefficient, with a minimum acceptable value of 0.70.

### Ethical considerations

The study protocol was developed in accordance with the ethical principles of the Declaration of Helsinki (1964) and received ethical approval from the Research Ethics Committee at King Khalid University (approval number: ECM#2023-3238).

The survey was administered anonymously, and no names, identification numbers, or IP addresses were collected. Participation was voluntary, and respondents were free to withdraw at any time by closing the survey. Although anonymity was maintained to minimize social desirability bias, it remains possible that some participants provided more favorable responses toward AI due to perceived professional expectations.

## Results

A total of 117 pharmacists participated in the study, yielding a response rate of approximately 49.4%. Of these, 22 participants (18.8%) reported that they had not heard the term artificial intelligence (AI) prior to the survey. Most participants were female (*n* = 70, 59.8%). The majority of respondents were aged 25–30 years (*n* = 89, 76.1%) and identified as Saudi nationals (*n* = 91, 77.8%). A substantial proportion had 1–4 years of work experience (*n* = 81, 69.2%), and 56 participants (47.9%) reported spending less than 5 minutes with each patient. Additionally, 44 participants (37.6%) reported working fewer than 40 hours per week. Most pharmacists indicated that they had access to the internet (*n* = 100, 85.5%) and scientific drug information resources (*n* = 98, 83.8%) in their workplace (Table 1).

**Table 1:** Baseline demographic and professional characteristics of participating community pharmacists in the Aseer region (*N* = 117).

**Characteristics**	**Category**	***n***	**%**
**Gender**	Male	47	40.2
	Female	70	59.8
**Age (years)**	25–30	89	76.1
	31–35	21	17.9
	36–40	7	6.0
**Nationality**	Saudi	91	77.8
	Non-Saudi	26	22.2
**Years of Experience**	1–4	81	69.2
	5–10	23	19.7
	11–15	13	11.1
**Number of daily pharmacy visitors**	<10	22	18.8
	10–20	28	24
	>20	67	57.2
**Number of working pharmacists**	<5	72	61.5
	5–10	33	28.2
	>10	12	10.3
**Number of Daily Prescriptions**	<5	23	19.7
	5–15	45	38.4
	>15	49	41.9
**Time spent with patients (minutes)**	<5	56	47.9
	5–10	47	40.1
	10–15	14	12.0
**Weekly working hours**	<40	44	37.6
	40–50	42	35.9
	>50	31	26.5
**Internet access in pharmacy**	Yes	100	85.5
	No	17	14.5
**Scientific drug information source available**	Yes	98	83.8
	No	19	16.2
**Heard About AI Before Survey**	Yes	95	81.2
	No	22	18.8

### Knowledge of artificial intelligence

Only the 95 participants (81.2%) who reported prior awareness of AI were included in the subsequent analyses. Among these pharmacists, approximately half (*n* = 49, 51.6%) believed they had sufficient knowledge of AI. More than half of respondents (*n* = 56, 58.9%) did not believe that AI would replace pharmacists within the healthcare system. However, a substantial majority (*n* = 68, 71.6%) believed that AI could help reduce errors in medical practice (Table 2).

**Table 2:** Community pharmacists’ self-reported knowledge of artificial intelligence and perceptions of its role in healthcare and error reduction (*N* = 117).

**Characteristics**	**N (%)**
**Do you think you have enough knowledge about Artificial Intelligence application/machine learning?**	
Yes	49 (51.6)
No	46 (48.4)
**Do you think that Artificial intelligence will replace the pharmacist, in the healthcare system?**	
Yes	39 (41.1)
No	56 (58.9)
**Do you think that “Artificial intelligence” will reduces errors in medical practice?**	
Yes	68 (71.6)
No	27 (28.4)

### Preferences for artificial intelligence applications

Pharmacists reported high levels of preference for various AI applications in pharmacy practice. The strongest support was observed for applications involving medical data collection, identifying drug-related problems, and medication dispensing. AI was also widely accepted for functions such as improving patient adherence, evaluating treatment options, and supporting clinical decision-making. Comparatively lower, although still positive, preferences were reported for applications involving detecting undiagnosed diseases and patient monitoring. Overall, respondents appeared most receptive to AI technologies that support data management and clinical decision support tasks (Table 3).

**Table 3:** Preferences of community pharmacists toward the use of artificial intelligence across various pharmacy practice activities (*N* = 95). Community pharmacists prefernces toward the use of artificial intelligence across various pharmacy practice activities, presenting response distributions, mean scores, and skewness.

**Item**	**Strongly disagree n (%)**	**Disagree n (%)**	**Neutral n (%)**	**Agree n (%)**	**Strongly agree n (%)**	**Mean ± SD**	**Skewness**
Medical data collection	1 (1.1)	4 (4.2)	13 (13.7)	44 (46.3)	33 (34.7)	4.10 ± 0.79	−0.73
Social data collection	2 (2.1)	6 (6.3)	18 (18.9)	41 (43.2)	28 (29.5)	3.90 ± 0.85	−0.46
Data claim (insurance)	3 (3.2)	7 (7.4)	20 (21.1)	40 (42.1)	25 (26.3)	3.81 ± 0.82	−0.48
Detecting hidden and undiagnosed diseases	4 (4.2)	10 (10.5)	23 (24.2)	35 (36.8)	23 (24.2)	3.66 ± 0.90	−0.30
Identifying drug-related problems	2 (2.1)	5 (5.3)	18 (18.9)	42 (44.2)	28 (29.5)	3.92 ± 0.79	−0.82
Specifying treatment outcome	3 (3.2)	6 (6.3)	21 (22.1)	41 (43.2)	24 (25.3)	3.81 ± 0.81	−0.63
Evaluating different treatment options	3 (3.2)	7 (7.4)	20 (21.1)	42 (44.2)	23 (24.2)	3.79 ± 0.83	−0.61
Designing care plan	3 (3.2)	8 (8.4)	21 (22.1)	41 (43.2)	22 (23.2)	3.75 ± 0.85	−0.55
Resolving drug-related problems	2 (2.1)	6 (6.3)	18 (18.9)	43 (45.3)	26 (27.4)	3.89 ± 0.80	−0.76
Follow-up and monitoring patients	4 (4.2)	9 (9.5)	25 (26.3)	36 (37.9)	21 (22.1)	3.63 ± 0.88	−0.38
Connecting healthcare provider systems	3 (3.2)	8 (8.4)	23 (24.2)	38 (40.0)	23 (24.2)	3.74 ± 0.84	−0.50
Obtain primary care and communicate with providers from home	4 (4.2)	9 (9.5)	24 (25.3)	36 (37.9)	22 (23.2)	3.66 ± 0.88	−0.36
Improving patient adherence	3 (3.2)	7 (7.4)	21 (22.1)	40 (42.1)	24 (25.3)	3.79 ± 0.82	−0.59
Medication dispensing	2 (2.1)	6 (6.3)	19 (20.0)	41 (43.2)	27 (28.4)	3.90 ± 0.80	−0.77
Patient counseling	3 (3.2)	8 (8.4)	22 (23.2)	39 (41.1)	23 (24.2)	3.75 ± 0.83	−0.52

### Attitudes toward artificial intelligence

Pharmacists generally demonstrated a moderately positive attitude toward artificial intelligence applications in pharmacy practice. The highest levels of agreement were observed for statements indicating that pharmacists prefer to stay updated with AI applications and would like to receive training on AI to improve their professional skills. Respondents also expressed agreement that AI could improve pharmacy services and patient outcomes. However, responses to the statement expressing concern that AI could replace pharmacists’ jobs were more neutral, indicating some apprehension regarding the potential impact of AI on the profession (Table 4).

**Table 4:** Attitudes of community pharmacists toward the implementation of artificial intelligence in community pharmacy practice (*N* = 95). Community pharmacists attitudes toward the implementation of artificial intelligence in pharmacy practice, including perceptions of benefits, concerns, and willingness to adopt AI-related training and applications.

**Item**	**Strongly disagree n (%)**	**Disagree n (%)**	**Neutral n (%)**	**Agree n (%)**	**Strongly agree n (%)**	**Mean ± SD**	**Skewness**
I like to be up-to-date in AI application in pharmacy setting	2 (2.1)	5 (5.3)	20 (21.1)	42 (44.2)	26 (27.4)	3.67 ± 0.65	−1.76
I like to receive training on AI because it is important to improve my career as a community pharmacist	2 (2.1)	4 (4.2)	18 (18.9)	45 (47.4)	26 (27.4)	3.68 ± 0.66	−1.84
I believe that AI will improve the services provided in the community pharmacy	3 (3.2)	6 (6.3)	23 (24.2)	39 (41.1)	24 (25.3)	3.50 ± 0.72	−1.10
I fear that AI could replace my job as pharmacist	8 (8.4)	16 (16.8)	29 (30.5)	25 (26.3)	17 (17.9)	3.11 ± 0.83	−0.21
I believe that AI applications will be widely used in pharmacy practice	2 (2.1)	6 (6.3)	21 (22.1)	41 (43.2)	25 (26.3)	3.62 ± 0.58	−1.28
I feel that AI can improve patient outcomes	2 (2.1)	7 (7.4)	19 (20.0)	43 (45.3)	24 (25.3)	3.57 ± 0.63	−1.34
I believe that AI is very useful for organizing our daily work	3 (3.2)	6 (6.3)	22 (23.2)	42 (44.2)	22 (23.2)	3.55 ± 0.67	−1.12
I believe that AI can multitask more effectively than humans and analyze data more quickly	2 (2.1)	7 (7.4)	21 (22.1)	40 (42.1)	25 (26.3)	3.59 ± 0.66	−1.18
I feel that AI can reduce the cost of care	4 (4.2)	9 (9.5)	27 (28.4)	34 (35.8)	21 (22.1)	3.36 ± 0.76	−0.74

### Association between participant characteristics and AI attitudes and preferences

Most demographic variables, including gender, age, nationality, years of experience, number of pharmacy visitors, number of daily prescriptions, and weekly working hours, were not significantly associated with either attitude or preference scores.

However, several factors were significantly associated with preference for AI applications. Higher preference scores were observed among pharmacists working in pharmacies with a greater number of pharmacists, those with internet access, those with scientific drug information sources, and those who reported shorter consultation times with patients. In contrast, attitude scores were significantly associated only with the belief that AI could reduce medical errors, suggesting that perceptions of AI’s clinical benefits may influence pharmacists’ attitudes toward its adoption (Table 5).

**Table 5:** Factors associated with community pharmacists preference and attitude scores toward adopting artificial intelligence in pharmacy practice (*N* = 95).

**Characteristics**	**Preference score Mean ± SD**	***P*-value**	**Attitude score Mean ± SD**	***P*-value**
**Gender**				
Male	3.79 ± 0.60	0.692^a^	3.44 ± 0.43	0.518^a^
Female	3.84 ± 0.55		3.50 ± 0.41	
**Age**				
25–30	3.78 ± 0.60	0.887^b^	3.46 ± 0.41	0.912^b^
31–35	3.80 ± 0.59		3.43 ± 0.45	
36–40	3.85 ± 0.49		3.47 ± 0.37	
**Nationality**				
Saudi	3.82 ± 0.57	0.311^a^	3.46 ± 0.42	0.722^a^
Non-Saudi	3.70 ± 0.61		3.43 ± 0.43	
**Years of experience**				
1–4	3.79 ± 0.58	0.821^b^	3.45 ± 0.44	0.603^b^
5–10	3.76 ± 0.61		3.50 ± 0.41	
11–15	3.88 ± 0.52		3.47 ± 0.39	
**Number of daily pharmacy visitors**				
<10	3.74 ± 0.64	0.754^b^	3.42 ± 0.46	0.448^b^
10–20	3.81 ± 0.55		3.44 ± 0.41	
>20	3.80 ± 0.58		3.50 ± 0.39	
**Number of working pharmacists**				
<5	3.71 ± 0.59	0.018^b^	3.41 ± 0.42	0.211^b^
5–10	3.84 ± 0.53		3.47 ± 0.40	
>10	4.21 ± 0.48		3.62 ± 0.36	
**Number of daily prescriptions**				
<5	3.72 ± 0.63	0.412^b^	3.50 ± 0.44	0.553^b^
5–15	3.75 ± 0.60		3.40 ± 0.46	
>15	3.85 ± 0.54		3.46 ± 0.39	
**Time spent with patients (minutes)**				
<5	3.83 ± 0.56	0.039^b^	3.44 ± 0.41	0.118^b^
5–10	3.78 ± 0.55		3.49 ± 0.42	
10–15	3.60 ± 0.71		3.30 ± 0.50	
**Weekly working hours**				
<40	3.82 ± 0.57	0.873^b^	3.50 ± 0.41	0.768^b^
40–50	3.80 ± 0.53		3.43 ± 0.43	
>50	3.77 ± 0.61		3.46 ± 0.41	
**Internet access**				
Yes	3.74 ± 0.57	0.002^a^	3.43 ± 0.42	0.074^a^
No	4.26 ± 0.49		3.62 ± 0.37	
**Scientific drug information sources**				
Yes	3.72 ± 0.55	0.001^a^	3.44 ± 0.42	0.264^a^
No	4.29 ± 0.50		3.55 ± 0.40	
**Enough knowledge about AI/ML**				
Yes	3.71 ± 0.60	0.683^a^	3.47 ± 0.43	0.398^a^
No	3.80 ± 0.53		3.43 ± 0.41	
**AI will replace pharmacists**				
Yes	3.75 ± 0.58	0.217^a^	3.40 ± 0.44	0.332^a^
No	3.85 ± 0.56		3.48 ± 0.41	
**AI reduces medical errors**				
Yes	3.79 ± 0.57	0.446^a^	3.39 ± 0.39	0.027^a^
No	3.71 ± 0.62		3.62 ± 0.44	

**Notes.**

a Independent *t*-test performed.

b one-way ANOVA. Statistically significant at *P* value ≤ 0.05.

### Reliability of the measurement scales

Both the 15-item preference scale and the 9-item attitude scale demonstrated good internal consistency, with Cronbach’s alpha values exceeding the acceptable threshold of 0.70. The preference scale had a mean score of 3.28 ± 0.66 and a Cronbach’s alpha of 0.89, while the attitude scale had a mean score of 3.67 ± 0.62 and a Cronbach’s alpha of 0.78 (Table 6).

**Table 6:** Internal consistency reliability of the preference and attitude scales measuring artificial intelligence adoption among community pharmacists (*N* = 95).

**Scale**	**Items**	**Mean**	**SD**	**Cronbach alpha**
Preference	15	3.28	0.66	0.892
Attitude	9	3.67	0.62	0.728

## Discussion

Artificial intelligence (AI) has been increasingly recognized as a powerful tool for enhancing healthcare delivery; however, its widespread implementation in pharmacy practice has not yet been fully realized (Al Meslamani, 2023). Among healthcare professionals, community pharmacists represent one of the most accessible healthcare providers, offering services that extend beyond medication dispensing to include health education, patient counseling, and preventive care (Jirjees et al., 2022). AI technologies have the potential to transform community pharmacy practice by supporting pharmacists in clinical decision-making, workflow management, and medication-related problem identification (Al Meslamani, 2023).

Accordingly, this study aimed to explore the current status of AI in the Aseer region of Saudi Arabia by examining community pharmacists’ knowledge, attitudes, and preferences toward AI implementation, as well as the factors influencing these perceptions. The goal was to identify potential gaps and challenges that may affect the integration of AI technologies into community pharmacy practice and to highlight opportunities for improving pharmacists’ professional performance. To our knowledge, this study represents the first investigation focusing on community pharmacists in the Aseer region. Only two previous studies in the Middle East have specifically evaluated AI perceptions among community pharmacists, conducted in Jordan and Riyadh, Saudi Arabia (Jarab et al., 2023; Syed & Al-Rawi, 2023a). Other studies in the region have mainly explored AI perceptions among other healthcare professionals and medical students (Abuzaid et al., 2021; Ahmed et al., 2022; Karan-Romero, Salazar-Gamarra & Leon-Rios, 2023; Qurashi et al., 2021; Swed et al., 2022).

In the present study, 81.2% of community pharmacists reported being aware of AI prior to the survey, indicating a relatively high level of awareness. However, only about half of these participants (51.6%) reported having sufficient knowledge about AI applications. Although this awareness level is lower than the 94.5% reported among community pharmacists in Riyadh (Syed & Al-Rawi, 2023a), it is higher than the 73.9% awareness reported among senior pharmacy students at King Saud University (Syed & Al-Rawi, 2023b). These differences may be attributed to variations in professional experience, exposure to digital health technologies, and the availability of AI-related training opportunities. The relatively limited knowledge reported by many participants in our study may also explain the cautious attitudes toward AI observed among pharmacists.

Regarding preferences for AI applications, pharmacists demonstrated moderate acceptance of implementing AI technologies in pharmacy practice, although preferences varied across different applications. The proportion of agreement across preference items ranged from approximately 34% to 58%, indicating variability in pharmacists’ perceived usefulness of specific AI tools. Similar findings were reported in Jordan, where 50.9% of community pharmacists expressed willingness to implement AI technologies in pharmacy practice (Jarab et al., 2023). In our study, pharmacists showed higher preference for AI applications related to medical data collection, identifying and resolving drug-related problems, medication dispensing, and improving patient adherence.

Conversely, lower preference was observed for AI applications involving detecting hidden or undiagnosed diseases and patient follow-up. This finding may reflect pharmacists’ perceptions regarding their scope of practice within community pharmacy settings. In the Aseer region, community pharmacy services traditionally focus on medication dispensing, patient counseling, and logistical tasks rather than diagnostic support or complex clinical decision-making. Consequently, pharmacists may perceive diagnostic AI tools as more aligned with physicians’ roles or as requiring advanced healthcare infrastructure that may not yet be widely available in routine pharmacy practice. Additionally, concerns regarding medico-legal responsibility and the accuracy of AI-generated clinical recommendations may contribute to more cautious perceptions in these areas.

Interestingly, Jordanian pharmacists demonstrated the strongest preference for AI tools that enhance communication with healthcare providers and access to primary care services from home (82.3%) (Jarab et al., 2023). While such applications represent one of the potential advantages of AI in community pharmacy, our findings suggest that pharmacists in the Aseer region currently perceive AI primarily as a supportive tool for workflow organization and medication-related decision support, rather than as a system for broader healthcare integration (Raza et al., 2022). This observation may also reflect the gradual evolution of clinical pharmacy practice in Saudi Arabia over the past decade (Badreldin, Alosaimy & Al-jedai, 2020).

With respect to attitudes toward AI, pharmacists in this study demonstrated a moderately positive overall attitude, with a mean attitude score of 3.67 ± 0.62. Several individual items revealed interest in remaining up to date with AI developments and receiving training on AI-enabled tools, suggesting a willingness among pharmacists to engage with emerging technologies. Approximately half of the participants agreed that AI could improve services provided in community pharmacies, would be widely used in pharmacy practice, and could analyze data more efficiently than humans. Furthermore, 71.6% of pharmacists believed that AI could reduce errors in medical practice, highlighting recognition of AI’s potential clinical benefits.

Previous studies among healthcare professionals have generally reported positive attitudes toward AI adoption (Abuzaid et al., 2021; Ahmed et al., 2022; Karan-Romero, Salazar-Gamarra & Leon-Rios, 2023; Qurashi et al., 2021; Swed et al., 2022). Similarly, more favorable perceptions toward AI integration in pharmacy practice were reported among community pharmacists in Riyadh and Jordan (Jarab et al., 2023; Syed & Al-Rawi, 2023a). However, the slightly more cautious attitudes observed in our study may reflect differences in regional exposure to digital health technologies, infrastructure availability, and professional training opportunities.

The results also indicated that 41.1% of participants believed that AI could replace pharmacists in the healthcare system, which is higher than the 25.6% reported among pharmacists in Riyadh (Syed & Al-Rawi, 2023a). This difference may be influenced by varying levels of exposure to AI technologies, as well as differences in workplace environments. It is also possible that pharmacists who are aware of AI may perceive its rapid technological advancement as a potential threat to professional roles.

In terms of factors influencing perceptions toward AI, the present study found that attitude toward AI was significantly associated with the belief that AI could reduce errors in medical practice. Certain workplace characteristics also influenced preference for AI adoption, including the number of pharmacists working in the pharmacy, availability of internet access, presence of scientific drug information resources, and consultation time with patients. However, demographic variables such as gender, age, nationality, and years of experience were not significantly associated with attitude or preference scores.

In contrast, Jarab et al. (2023) reported that weekly working hours and knowledge of AI technology were significantly associated with willingness and attitudes toward AI, while years of experience, income, and daily pharmacy visitor numbers were associated with willingness to implement AI. The differences between these findings and those of the present study may partly reflect the characteristics of our study sample, where 69.2% of pharmacists had only 1–4 years of professional experience. Pharmacists with limited experience may be less familiar with the workload demands of routine pharmacy tasks, which could influence how they perceive the potential benefits of AI technologies.

Practical barriers may also influence pharmacists’ perceptions of AI adoption. Community pharmacies in the Aseer region operate under varying levels of digital infrastructure, with some locations still relying on basic pharmacy information systems. Limited access to high-speed internet, advanced hardware, or integrated electronic health record systems may reduce the perceived feasibility of implementing AI tools in daily workflows. Additionally, heavy workloads, staffing limitations, and a focus on high-volume dispensing may restrict pharmacists’ opportunities to engage with new technologies or attend AI-related training programs.

These findings should be interpreted within the context of the study population and the regional practice environment. Digital health adoption and AI implementation are more advanced in major Saudi cities such as Riyadh and Jeddah compared with some areas in the Aseer region. Therefore, the attitudes and preferences observed in this study may not be fully generalizable to pharmacists practicing in other regions of Saudi Arabia. Future research involving multiple regions across the country would provide a more comprehensive understanding of pharmacists’ perceptions toward AI and help guide national strategies for AI integration in pharmacy practice.

Overall, our findings suggest that community pharmacists in the Aseer region demonstrate moderate awareness, cautious attitudes, and selective preferences toward AI implementation. Differences in perceptions across Middle Eastern countries and within Saudi regions highlight the importance of broader national assessments to support effective AI adoption strategies in pharmacy practice.

## Conclusion

This study provides initial insights into AI awareness, attitudes, and preferences among community pharmacists in the Aseer region of Saudi Arabia. Although most pharmacists were aware of AI, only about half reported sufficient knowledge regarding its applications. Pharmacists demonstrated moderate preference for AI implementation and moderately positive attitudes toward its use, particularly for applications supporting medication-related tasks and workflow management.

To support the responsible integration of AI into community pharmacy practice, policymakers and professional organizations should prioritize structured training programs on AI-enabled tools, incorporate AI-related content into undergraduate pharmacy curricula, and invest in digital infrastructure that enables safe and effective AI utilization. Pharmacy managers can facilitate this transition by providing access to reliable decision-support systems, encouraging participation in AI-related professional development, and ensuring that AI is positioned as a supportive technology that enhances, rather than replaces, pharmacists’ clinical expertise.

Further multi-regional research across Saudi Arabia is needed to better understand geographic variations in AI adoption and to support the development of coordinated national strategies for integrating AI technologies into pharmaceutical care.

### Study Limitations

The present study has several limitations that should be acknowledged. First, although the target sample size was 237 pharmacists to ensure sufficient statistical power, only 117 ultimately participated. This smaller-than-expected sample may have resulted in reduced statistical power, limiting the ability to detect significant associations for some variables. Second, the exclusion of pharmacists who had not previously heard of artificial intelligence (AI) could have introduced sampling bias. By restricting participation to individuals with at least minimal prior exposure to AI concepts, the study may have overestimated positive attitudes and preferences toward AI, thereby limiting the generalizability of the findings to the wider population of pharmacists.

Third, AI knowledge was measured using a single self-rated item, which may not accurately represent actual levels of understanding. Self-assessment measures are inherently subjective and may not capture the depth or accuracy of respondents’ knowledge. Future research should consider incorporating multi-item, objective assessments—such as scenario-based questions or standardized knowledge tests—to produce a more valid and reliable measure of AI knowledge among pharmacists.

Lastly, while the questionnaire underwent face and content validity review by academic experts, internal consistency reliability (*e.g.*, Cronbach’s alpha) was calculated after data collection and not during a pilot phase prior to recruitment. This omission may limit confidence in the reliability of the attitudinal and preference scales used in the current study. Accordingly, future research should conduct a pilot study and report reliability coefficients to enhance the robustness of the instrument. Conducting a nationwide cross-sectional study that includes community pharmacists with varying years of experience across all 13 provinces of Saudi Arabia is also recommended to ensure broader representativeness and more generalizable findings.

##  Supplemental Information

10.7717/peerj.21246/supp-1 Supplemental Information 1 All collected coded variables and participant responses used for analysis

10.7717/peerj.21246/supp-2 Supplemental Information 2 Raw Data Rows represent observations, and columns represent variables (defined in the header ) . Data collection and preprocessing details are described in the Methods section.

10.7717/peerj.21246/supp-3 Supplemental Information 3 STROBE checklist

10.7717/peerj.21246/supp-4 Supplemental Information 4 Questionnaire

## Additional Information and Declarations

### Data Availability

The following information was supplied regarding data availability: The raw data is available in the Supplemental Files.
