# Supplementary material for: Navigating the future—community pharmacists’ attitudes towards AI integration in pharmacy services: a cross-sectional study in Aseer, Saudi Arabia
Source: PeerJ. 2026 May 8;14:e21246. doi: 10.7717/peerj.21246 (PMC13159730; doi:10.7717/peerj.21246)
Supplement: Supplemental Information 4 [file peerj-14-21246-s004.pdf]

We are a group of academic researchers conducting a research project on the "Artificial Intelligence in Pharmacy Practice in Asser Region"

The participant must be aged≥18 years and community pharmacist .
Your participation in this research project is voluntary.
You may choose not to participate.
If you decide to participate in this research, you may withdraw at any time.
The procedure involves filling a survey that will take approximately 5 minutes. We ensure that your responses will be confidential.

Demographical Information

1. Age

Mark only one oval.

- 25 - 30
- 31 - 35
- 36 - 40
- > 40

2. Sex

Mark only one oval.

- Male
- Female

3. Nationality

Mark only one oval.

- Saudi
- Non Saudi

4. Years of experience

Mark only one oval.

- 1-4
- 5-10
- 11 - 15
- > 15

5. Number of daily pharmacy visitors

Mark only one oval.

- < 10
- 10 - 20
- > 20

6. Number of working pharmacists

Mark only one oval.

- < 5
- 5 -10
- > 10

7. Number of daily prescriptions

Mark only one oval.

- < 5
- 5 - 10
- 10 - 15
- >15

8. Weekly working hours

Mark only one oval.

- < 40
- 40 - 50
- > 50

9. Internet access

Mark only one oval.

- Yes
- No

10. Drug information source

Mark only one oval.

- Yes
- No

11. Average time spent with the patient

Mark only one oval.

- < 5 min
- 5 - 10 min
- 10 - 15 min
- > 15 min

Knowledge

12. Have you heard the term (Artificial Intelligence) before this survey ?

Mark only one oval.

- Yes
- No

13. Do you think you have enough knowledge about Artificial Intelligence application/machine learning ?

Mark only one oval.

- Yes
- No

14. Do you think that Artificial intelligence will replace the pharmacist, in the healthcare systen

Mark only one oval.

- Yes
- No

15. Do you think that "Artificial intelligence" will reduces errors in medical practice ?

Mark only one oval.

- Yes
- No

Willingness

Are you able to apply AI in the following practices

16. Medical data collection

Mark only one oval.

- Strongly agree
- Agree
- Neutral
- Disagree
- Strongly Disagree

17. Social data collection

Mark only one oval.

- Strongly agree
- Agree
- Neutral
- Disagree
- Strongly Disagree

18. Data claim (insurance)

Mark only one oval.

- Strongly agree
- Agree
- Neutral
- Disagree
- Strongly Disagree

19. Detecting hidden and undiagnosed diseases

Mark only one oval.

- Strongly agree
- Agree
- Neutral
- Disagree
- Strongly Disagree

20. Identifying drug-related problem

Mark only one oval.

- Strongly agree
- Agree
- Neutral
- Disagree
- Strongly Disagree

21. Specifying treatment outcome

Mark only one oval.

- Strongly agree
- Agree
- Neutral
- Disagree
- Strongly Disagree

22. Evaluating different treatment options

Mark only one oval.

- Strongly agree
- Agree
- Neutral
- Disagree
- Strongly Disagree

23. Designing care plan

Mark only one oval.

- Strongly agree
- Agree
- Neutral
- Disagree
- Strongly Disagree

24. Resolved drug-related problem

Mark only one oval.

- Strongly agree
- Agree
- Neutral
- Disagree
- Strongly Disagree

25. Follow up and monitoring patients

Mark only one oval.

- Strongly agree
- Agree
- Neutral
- Disagree
- Strongly Disagree

26. Connecting healthcare provider systems

Mark only one oval.

- Strongly agree
- Agree
- Neutral
- Disagree
- Strongly Disagree

27. Obtain primary care and communicate with care providers from home

Mark only one oval.

- Strongly agree
- Agree
- Neutral
- Disagree
- Strongly Disagree

28. Improving patient adherence

Mark only one oval.

- Strongly agree
- Agree
- Neutral
- Disagree
- Strongly Disagree

29. Medication dispensing

Mark only one oval.

- Strongly agree
- Agree
- Neutral
- Disagree
- Strongly Disagree

30. Patient counseling

Mark only one oval.

- Strongly agree
- Agree
- Neutral
- Disagree
- Strongly Disagree

Attitude

31. I like to be up-to-date in AI application in pharmacy setting

Mark only one oval.

- Strongly Agree
- Agree
- Neutral
- Disagree
- Strongly Disagree

32. I like to receive training on AI because it's important to improve my career as a community pharmacist

Mark only one oval.

- Strongly Agree
- Agree
- Neutral
- Disagree
- Strongly Disagree

33. I believe that AI will improve the services provided in the community pharmacy

Mark only one oval.

- Strongly Agree
- Agree
- Neutral
- Disagree
- Strongly Disagree

34. I fear that AI could replace my job as pharmacist

Mark only one oval.

- Strongly Agree
- Agree
- Neutral
- Disagree
- Strongly Disagree

35. I believe that AI applications will be widely used in pharmacy practice

Mark only one oval.

- Strongly Agree
- Agree
- Neutral
- Disagree
- Strongly Disagree

36. I feel that AI can improve patient outcomes

Mark only one oval.

- Strongly Agree
- Agree
- Neutral
- Disagree
- Strongly Disagree

37. I believe that AI is very useful for organizing our daily work

Mark only one oval.

- Strongly Agree
- Agree
- Neutral
- Disagree
- Strongly Disagree

38. I believe that AI can multitask more effectively than humans and analyze data more quickl

Mark only one oval.

- Strongly Agree
- Agree
- Neutral
- Disagree
- Strongly Disagree

39. I feel that AI can reduce the cost of care

Mark only one oval.

- Strongly Agree
- Agree
- Neutral
- Disagree
- Strongly Disagree

40. Data Collector Number

Mark only one oval.

- 1
- 2
- 3
- 4
- 5
- 6

This content is neither created nor endorsed by Google.

Google Forms
